# The Relationship between Mortality from Cardiovascular Diseases and Total Drinking Water Hardness: Systematic Review with Meta-Analysis

**DOI:** 10.3390/foods12173255

**Published:** 2023-08-29

**Authors:** Aleksandra Bykowska-Derda, Marcin Spychala, Magdalena Czlapka-Matyasik, Mariusz Sojka, Jerzy Bykowski, Mariusz Ptak

**Affiliations:** 1 Department of Human Nutrition and Dietetics, Poznan University of Life Sciences, Wojska Polskiego 31, 60-624 Poznan, Poland; aleksandra.derda@up.poznan.pl (A.B.-D.); magdalena.matyasik@up.poznan.pl (M.C.-M.); 2 Department of Hydraulic and Sanitary Engineering, Poznan University of Life Sciences, Piątkowska 94A, 60-649 Poznan, Poland; marcin.spychala@up.poznan.pl; 3 Department of Land Improvement, Environmental Development and Spatial Management, Poznań University of Life Sciences, Piątkowska 94E, 60-649 Poznan, Poland; mariusz.sojka@up.poznan.pl (M.S.); jerzy.bykowski@up.poznan.pl (J.B.); 4 Department of Hydrology and Water Management, Adam Mickiewicz University, Krygowskiego 10, 61-680 Poznan, Poland

**Keywords:** water hardness, arterial sclerosis, hypertension, heart

## Abstract

Background: Interest in water chemical activity, its content, and its impact on human health has greatly increased throughout the last decade. Some studies suggest that drinking water with high hardness may have preventative effects on cardiovascular diseases. This study aims to investigate the association between drinking water hardness and cardiovascular disease (CVD) mortality. Methods: The study selection process was designed to find the association between drinking water hardness and CVDs mortality. The search included both qualitative and quantitative research and was performed in three databases: Web of Science (Clarivate Analytics, Ann Arbor, MI, USA), PubMed (National Institute of Health, Bethesda, MA, USA), and Scopus (Elsevier, RELX Group plc, London, UK). The project was registered in the International Prospective Register of Systematic Reviews (PROSPERO), registration number: CRD42020213102. Results: Seventeen studies out of a total of twenty-five studies qualitatively analyzed indicated a significant relation between total water hardness and protection from CVD mortality. The quantitative analysis concluded that high drinking water hardness has a significantly lowering effect on mortality from CVDs, however, the heterogeneity was high. Conclusions: This systematic literature review shows that total water hardness could affect CVD prevention and mortality. Due to the many confounding factors in the studies, more research is needed.

## 1. Introduction

Cardiovascular diseases (CVDs) are the leading cause of death worldwide, taking almost 18 million lives each year [1]. According to the World Health Organization (WHO), more than four out of five CVD deaths are due to heart attacks and strokes, and one-third of these deaths occur prematurely in people under 70 years of age. There are multiple factors known to be responsible for the etiology of CVDs; the most prominent risks are highly processed foods, unhealthy diet, physical inactivity, and tobacco or alcohol use [1]. Even though knowledge of these factors is widespread and prevention of non-communicable disease is a highly researched topic, the problem persists. Based on scientific research, the prevention of CVDs includes the cessation of tobacco use, reduction of salt in the diet, eating more fruit and vegetables, regular physical activity, and avoiding the use of alcohol. However, these recommendations do not include the “most forgotten” nutrient: water and its quality.

Interest in water chemical activity, its content, and its impact on human health has been intensively increasing throughout recent decades.

An analysis of water quality in Limpopo province showed that periodic interruptions in the municipal water supply and certain water transportation and storage practices increase the risk of water contamination [2]. Mining activities in the southern part of Armenia affect the quality of life of the region’s residents in terms of the use of river water contaminated with heavy metals for irrigation, as well as the oral and dermal effects of drinking water on humans [3]. Studies of *E. coli* bacteria in water in Kenya indicate that water treatment is particularly important after heavy rainfall and high temperatures [4]. In the case of the West Bank of Palestine, an assessment of the microbiological and physicochemical quality parameters of drinking water allows us to conclude that only a small portion of the samples analyzed (2%) required chlorination, while most sources were classified as non-hazardous [5].

Health recommendations for water consumption are commonly unfulfilled. Drinking water recommendations vary depending on food intake, climate temperature, medical conditions, and exercise intensity [6]. The inefficient supply of water is responsible for the risks of many diseases, e.g., fatal cases of ischemic heart disease, urinary tract infections, constipation, and kidney stones [7,8].

There have also been some reports suggesting that the mineral content of water could severely affect human health [9,10,11,12]. Water is a significant source of minerals including calcium and magnesium compounds in the diet. Thanks to their ionized forms, both calcium (Ca^2+^) and magnesium (Mg^2+^) may be more bio-available than some of the forms of these elements in food [10,13,14]. An adequate intake of Ca^2+^ and Mg^2+^ is also related to better health. For example, they play a role in muscle contraction or are a part of the skeletal system. Ca^2+^ and Mg^2+^ have recommended daily intakes, which seem difficult to obtain with current common dietary behaviors [15,16]. Consequently, low intake of those minerals is associated with CVDs [17,18]. Despite this data, nutrition professionals and scientists rarely address the quality of drinking water.

Ca^2+^ and Mg^2+^ are two cations commonly occurring in nature in the composition of sedimentary rocks. These cations have a basic share in total hardness, known as the ability of water to react with soap and form deposits, e.g., insoluble metals or salts [19]. Additionally, the multivalent ions aluminum, barium, iron, manganese, strontium, zinc, and hydrogen cations also constitute a small share in total hardness [19,20,21]. The WHO recommends drinking water hardness in the range of 50–500 mg/L CaCO_3_ [19]. 

There are a few case–control studies and reviews which suggest a protective role of drinking hard water in CVD incidence and mortality [19,22,23]. Most studies reviewed in [10] analyzed the content of single ions of calcium and magnesium. However, to the best knowledge of the authors, there has been no meta-analysis concerning total water hardness. CVD protection may not only be caused by a high intake of Ca^2+^ and Mg^2+^ but also by the corroding properties of water. Hard water is less corrosive, which prevents toxic elements in plumbing systems (such as lead) from entering the gastrointestinal tract. Low water hardness (softness) has also been suggested as being associated with cancer [9,24], diabetes or metabolic syndrome [25,26], neurological disorders, atrophy, lateral sclerosis, pre-eclampsia in pregnant women, high blood pressure [27], bone fractures, and bone development in children [14]. 

Hard water has been also associated with some negative health impacts, such as kidney stones [28] or the risk of eczema [29]. However, the data on kidney stones and drinking water is not clear. Cohort studies report that drinking hard water increased urinary calcium concentration, but not the rate of kidney stone formation [30]. It is not known if the long-term drinking of hard water in large quantities has an impact on kidney function. This could be problematic, especially for people with a high risk of kidney dysfunctions, for example, patients with diabetes. There are also many factors influencing the association between drinking hard water and kidney stones that should be further investigated. For example, the timing of drinking water with high mineral content between meals [31].

Despite many reports suggesting the relationship between [water] hardness, calcium and magnesium concentrations, and health conditions [32] including CVD mortality [10,12,20,33], the debate on this topic continues and is yet to demonstrate causality [19,34]. Certain factors influencing these results are caused by the variability in populations worldwide, healthcare systems, or water treatment types. CVD rates are different throughout the globe, mainly because of genetic predispositions and different lifestyles, physical activity, or dietary patterns. The Western dietary pattern, prominent in developed countries, has been highly associated with CVDs. This dietary pattern is characterized by the frequent intake of processed food high in sugar and saturated fats, and low intake of fruits, vegetables, and whole grain products [35].

Water quality also differs between countries. Europe has some of the most stringent rules on water quality, specified in the European Union Directive 98/83/EC of 3 November 1998 on the quality of water intended for human consumption. However, this Directive does not include information on water hardness [23]. Also, the water quality, total drinking water hardness, and the protective role against CVDs have not been fully investigated. Keeping in mind various studies associating water hardness and health, the information concerning water hardness should be clarified and summarized. Therefore, this study aims to investigate the association between drinking water hardness and CVD mortality rates.

## 2. Materials and Methods

**Figure 1:**
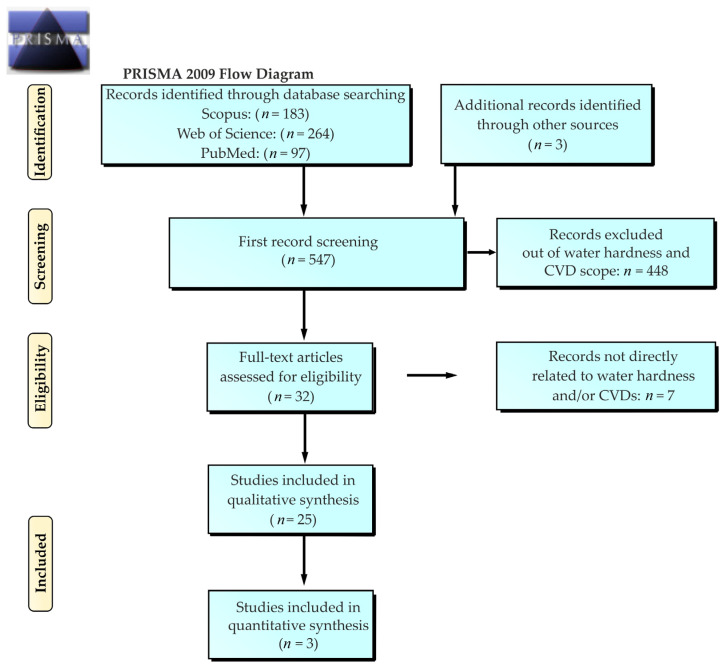
PRISMA flow diagram of the search process.

The project was registered in the International Prospective Register of Systematic Reviews (PROSPERO), registration number: CRD42020213102. The identification and screening process for selecting reviewed articles was performed according to the PRISMA (preferred reporting items for systematic reviews) guidelines and is presented in the diagram below (Figure 1).

### 2.1. Study Selection Process

The study selection process was designed to find the association between drinking water hardness and CVD mortality rates and was performed using three databases, the Web of Science (Clarivate Analytics, Ann Arbor, MI, USA), PubMed (National Institute of Health, Bethesda, MA, USA), and Scopus (Elsevier, RELX Group plc, London, UK), between October 2020 and February 2023. The strategy was based upon the following index terms, titles, or abstracts: (cardiovascular) OR (ISCHEMIC) OR (hypertension) OR (heart) OR (arterial sclerosis) AND (water hardness). The study selection process included an assessment of titles, abstracts, keywords, and full text, which were performed by two independent researchers in each database. At each step, all disagreements between the researchers were resolved after consultation with the review coordinator. In the case of a disagreement during the title assessment process, the paper was included in the next step. 

### 2.2. Inclusion and Exclusion Criteria 

The studies taken into consideration concerned the association between mortality from any CVD and drinking water hardness. For the narrative part of the review, articles were excluded under the following circumstances: (1) not an original research paper (short communication, review, meta-analysis, book chapter, etc.); (2) published in languages other than English; and (3) published before the year 1990.

Additionally, studies included in the meta-analysis had to include numbers of total hardness or calcium and magnesium data, and CVD mortality corresponding to each region. Hard versus soft water was defined according to the water hardness scale, where water containing calcium carbonate at concentrations below 60 mg/L is generally considered as soft; 60–120 mg/L as moderately hard; 120–180 mg/L, hard; and more than 180 mg/l as very hard [36]. 

### 2.3. Statistics

Meta-analysis was performed in the Cochrane Rev Man 5.4.1 (Cochrane.org, The Nordic Cochrane Centre, Copenhagen, Denmark). To perform the meta-analysis, the fixed Mantel–Haenszel test and inverse-variance method were used. The heterogeneity was interpreted as I2 where <25 % = low heterogeneity; 25–50% = moderate heterogeneity; and >75 % = high heterogeneity [37]. To summarize the results of the meta-analysis, it was presented in the form of a forest plot. To obtain total water hardness in the maximal amount of studies, it was calculated from total magnesium and calcium content by using the Keisan Online Calculator service (Casio Computer Co., Ltd., Tokyo, Japan). The study by Nagy et al. [38] had different regions and hard water values that were analyzed separately; therefore, it was also analyzed separately in our study (a and b). The study by Nerbrant et al. [39] further analyzed men and women separately. 

The search included both qualitative and quantitative methods in studies. A quality assessment of questionable articles was performed with a checklist described by Kmet et al. [40]. 

## 3. Results

**Figure 2:**
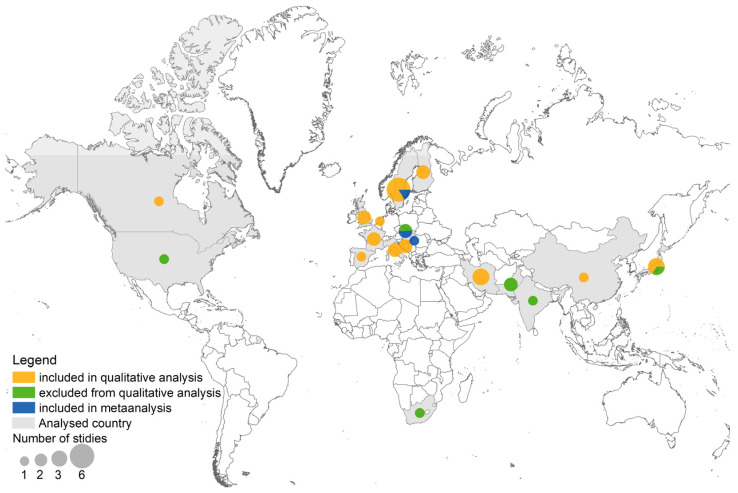
Studies included in the review according to the analyzed country.

**Figure 3:**
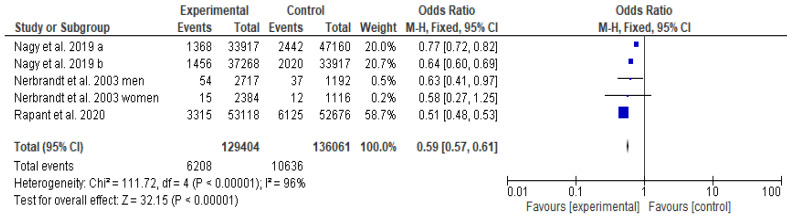
Forest plot analysis: the relationship between water hardness and CVDs [12,38,39].

Out of 547 studies analyzed by the first screening, 34 qualified for full-text screening (Figure 1 and Figure 2). Three studies were found from Iran [25,41,42], two from Japan [43,44], and one each from Canada [45], India [11], Taiwan [46], South Africa [47], Pakistan [48], and Samoa [49], respectively. There were 17 studies from European countries: Slovakia [12], Hungary [38], Bosnia and Herzegovina [22], Netherlands [50], United Kingdom [33,34], Sweden [39,51,52,53,54,55], Spain [56], Finland [57,58], France [13,59], Italy [60,61], and Serbia [62]. These studies differed in design. Seven ecological studies could not be included in the meta-analysis because of missing data (Table 1). Eventually, the quantitative analysis included three European studies (Table 2, the forest plot for those studies is depicted in Figure 2). According to the meta-analysis, the inhabitants of areas with hard water (experimental group) had lower CVD mortality [rates] than inhabitants of areas with soft water (control group). However, the heterogeneity of the studies was high; I^2^ = 96% (Figure 3). The lowest odds ratio and the most significant association were found in the study by Rapant et al. [12], and the highest in the subgroup of the study by Nagy et al. [38]. Out of the total 25 studies fully reviewed, eight had either no or a non-significant relationship between water hardness and protection from CVDs and/or CVD-related mortality. 

## 4. Discussion

This study aimed to analyze the association between drinking hard water and mortality rates from CVDs and was the first study to quantitatively analyze the total water hardness alongside CVD mortality rates. Drinking hard water, in this analysis, reduced mortality from CVDs by forty percent. Out of 25 studies analyzed qualitatively, a total of 28% of all studies reported no relationship or a non-significant relationship between water hardness and CVD prevention and mortality. 

### 4.1. Possible Effects of Drinking Hard Water on CVD Mortality

Drinking water with a high hardness seems to prevent mortality from CVDs. The study by Rapant et al. analyzed the four major causes of death (cardiovascular, oncological, gastrointestinal, and respiratory tract) among inhabitants drinking hard and soft water [12]. The study showed that inhabitants from areas with hard drinking water had a better health status overall and higher life expectancy than inhabitants from areas with low drinking water hardness [12]. Another ecological study, where the negative relationship between water hardness and CVD mortality was found, was performed by Nagy et al. [38]. Both of the studies [12,38] controlled for the quality of life but not for the dietary mineral intake or water filtration. Our meta-analysis also included a smaller study wherein individuals had their diet and water samples assessed [39]. This study concluded that calcium-rich water has a relation with CVD mortality, but surprisingly perhaps, not with magnesium or total water-hardness.

Our analysis also shows that calcium and magnesium content in water seem to have some association with CVD mortality rates [13,52,55,59,63]. As mentioned earlier, drinking water could be a valuable source of calcium and magnesium, which are hard to obtain in the average Western diet [16]. Magnesium plays a key role in cardiovascular health. Mg^2+^ ions take part in the modulation of ion transporters in the heart and, therefore, in the regulation of muscle tone, atherogenesis and thrombosis, vascular calcification, and proliferation of endothelial and vascular smooth muscles [64]. In turn, evidence of calcium’s role in CVD prevention has inconsistent results. On the one hand, high calcium dietary intake could have a positive role in the prevention of CVD, while on the other, very high calcium intake, mostly by dietary supplements, has either no or an adverse effect [65,66]. The Ca^2+^ downregulates the renin–angiotensin system and improves sodium–potassium balance [65]. Additionally, studies on rats show that a high intake of calcium could bind the fatty acids in the intestinal lumen and prevent their absorption, similar to a high-fiber diet [67]. Therefore, drinking water with high mineral content could have an impact on the prevention of non-communicable diseases. 

### 4.2. Hard Water and Prevention from CVDs

Our qualitative analysis also included studies that considered the influence of hard water on the morbidity of the population. Momeni et al. [42] found an inverse association between water hardness and the magnesium content in drinking water and the risk of CVD in Iran. Water hardness was also associated with a lower risk of hypertension [25]. Calcium water content, in turn, was associated with a lower risk of acute myocardial infarction [51] and ischemic heart disease [62].

An earlier meta-analysis from 2017 by Gianfredi et al. found that high concentrations of both calcium and magnesium seem to offer protection from CVDs, however, it did not analyze the total water hardness [10]. Our analysis supports a similar conclusion; that there were not enough homogenous studies and so, more studies concerning this topic are needed. Keeping in mind that soft water is preferable in households [68], nutrition professionals should consider the water quality of their patients and accordingly educate them about the possible health benefits of drinking hard water as opposed to soft water. 

### 4.3. Strengths and Limitations of the Review

The limitation of this review lies in the variability of and the existence of only observational types of studies. Therefore, more studies are needed that analyze the association between water hardness and CVD mortality. 

There are multiple factors already established influencing CVD mortality. Among others are lifestyle habits, diet, exercise, stress, male gender, and socioeconomic levels. the studies in our review have considered many of those factors. Gender plays a role in CVD mortality because of different life expectancies and the protective role of estrogens from vascular damage [69]. A few of the analyzed studies also differentiated and took into account gender demography [39,52,54,55]. Factors that also need to be considered are the quality of life, healthy nutrition, and dietary intake of calcium and magnesium, factors that the studies rarely included [25,34,39,51,54]. 

The level of urbanization is also a known factor influencing CVD occurrence. Inhabitants from urbanized areas have different lifestyles and quality of life than inhabitants from rural areas. It is not clear whether the urbanized or non-urbanized area is a [greater] risk factor for CVD and it varies among population studies in different areas around the world [70]. Most of the analyzed studies considered the level of urbanization, and one of them compared water hardness between urban and rural areas, which could influence the results [22]. 

The three included studies in the meta-analysis are from the European Union area (Hungary [38], Sweden [39], and Slovakia [12]), where the quality of water is highly regulated [23]. The study from Slovakia in particular had a high sample size [12]. The studies included in the meta-analysis only come from central Europe. Both calcium and magnesium intake seem to be inefficient in the Western diet, which is associated with CVD [17,18]. All of the studies included in the meta-analysis were adjusted for life quality and urbanization level. In the study by Nerbrandt et al. [39], participants were also asked about their dietary intake of calcium and magnesium as well as about using filtered water. The water used for drinking by the inhabitants could vastly influence the results of the studies. The households often use filtration systems or drink bottled water, because hard water tends to leave residues on household appliances. 

### 4.4. Studies with No or Non-Significant Effect on CVDs

Out of 25 analyzed studies, 8 of them did not show a significant relationship between water hardness and CVD mortality [33,34,39,41,43,44,50,56]. The studies had different designs and, while most were ecological studies [34,41,43,44,50,56], there were also time-series approaches investigating the changes in drinking water hardness [33] or on smaller sample sizes focused in more detail on individuals [39]. The differences in results may be explained by the diversity of factors influencing CVD mortality [71]. However, it is worth noting that all of the studies had at least a weak inverse association between hard water and CVD morbidity or mortality. None of the studies demonstrated any negative effect of hard water on CVDs, moreover, some studies showed an association between hard water and better health status. There is a need for more research on inter-individual variability in large population studies on specific markers of CVDs and drinking water hardness. 

### 4.5. Corrosive Properties of Soft Water and CVDs Health Considerations

It is important to remember that the total water hardness not only includes calcium and magnesium content but also other ions. In addition, hard water is less corrosive than soft water. Soft water has some positive effects on the conditions of the water supply installation - it does not leave sediments and, therefore, the inner light of the pipelines is not reduced. Such water does not irritate skin and is especially recommended for people who have skin problems such as various diseases, allergies, and hypersensitivity [72]. Soft water (under some conditions) is considered to be corrosive or even aggressive to water supply system material, e.g., steel or copper [73], especially in the case of older pipes, which do not have internal protection. Corrosion in soft water is complex and still not completely explained; it is, however, considered to be related to natural organic matter, chlorine, and temperature [74]. 

A rather rarely-raised but potentially serious problem, is the indirect influence of soft water on humans, resulting from the consumption of water containing increased concentrations of certain elements (e.g., metals such as copper or iron) released from pipes due to the corrosive effect of soft water [10]. Although copper and iron are important for proper functioning, they, like most compounds, are toxic in excess for humans. High iron stores seem to be associated with the serum lipid profile; however, they are not always related to dietary intake of iron [75]. Iron in the oxidative state is a highly reactive oxidative species (ROS) that may destroy different tissues and, therefore, lead to non-communicable diseases, including CVDs [75].

## 5. Conclusions

In conclusion, hard water seems to have a protective effect on mortality from CVDs. According to WHO, the recommended drinking-water hardness is between 50 and 500 mg/L CaCO_3_ [19]. Our analysis shows that water hardness within the upper limits of this range could influence CVD prevention and mortality rates. These results support the hypothesis that drinking hard water could assist in the prevention of CVDs. However, due to numerous confounding factors in the studies, this information should be taken with caution. There is more research needed, including intervention studies considering all the factors influencing CVD risks.

## Figures and Tables

**Table 1:** Studies excluded from the meta-analysis.

Study	Reason for Exclusion	Outcome
Miyake et al., 2003 [43] (Japan)	Ecological study	No relation between water hardness and mortality from cerebrovascular diseases.
Miyake et al., 2004 [44] (Japan)	Ecological study	No relation between water hardness and mortality from coronary heart disease.
Ferrandiz et al., 2004 [56] (Spain)	Ecological study	A non-significant inverse relationship between water hardness and mortality from CVDs.
Kousa et al., 2004 [57] (Finland)	Ecological study	An inverse relationship between water hardness and coronary heart disease.
Lake et al., 2010 [33] (United Kingdom)	Ecological study	No relation between water hardness from coronary heart disease mortality.
McLeod et al., 2018 [45] (Canada)	Ecological study	An inverse relationship between water hardness and magnesium levels and CVDs.
Hossienifar et al., 2019 [41] (Iran)	Ecological study	A non-significant inverse relationship between the water hardness and CVDs.
Dore et al., 2021 [61] (Italy)	Ecological study	An inverse relationship between water hardness and mortality from coronary artery disease.
Rosenlund et al. [51] 2005 (Sweden)	Prevention of CVDs	An inverse relationship between water calcium and acute myocardial infarction.
Momeni et al., 2014 [42] (Iran)	Prevention of CVDs	An inverse relationship between water hardness and magnesium levels and CVDs.
Knezovic et al., 2014 [22] (Bosnia and Herzegovina)	Prevention of CVDs	An inverse relationship between water hardness and CVDs.
Stevanovic et al., 2017 [62] (Serbia)	Prevention of CVDs	An inverse relationship between Ca and Mg from drinking water and risk factors for ischemic heart disease.
Yousefi et al., 2019 [25] (Iran)	Prevention of CVDs	An inverse relationship between water hardness and hypertension.
Helte et al., 2022 [54] (Sweden)	Prevention of CVDs	An inverse relationship between water magnesium and calcium content and risk of stroke in postmenopausal women.
Rylander et al., 1991 [53] (Sweden)	Data missing	An inverse relationship between water hardness and CVDs mortality.
Piispanen 1993 [58] (Finland)	Data missing	An inverse relationship between water hardness and CVDs mortality between the highest and lowest region but a small relationship nationwide.
Bernardi et al., 1995 [60] (Italy)	Data missing	An inverse relationship between water hardness and ischemic heart disease mortality.
Rubenowitz et al., 1996 [55] (Sweden)	Data missing	An inverse relation between calcium and magnesium in drinking water and mortality from acute myocardial infarction in men while comparing it with oncology patients.
Rubenowitz et al., 1999 [52] (Sweden)	Data missing	An inverse relation between calcium and magnesium in drinking water and mortality from acute myocardial infarction in women while comparing it with oncology patients.
Sauvant et al., 2000 [59] (France)	Data missing	An inverse relation between calcium in drinking water and mortality from CVDs.
Marque et al., 2003 [13] (France)	Data missing	An inverse relation between calcium in drinking water and mortality from CVDs.
Yang et al., 2006 [63] (Taiwan)	Data missing	An inverse relation between calcium in water and mortality from acute myocardial infarction.
Morris et al., 2008 [34] (United Kingdom)	Data missing	No relation between water hardness from CVDs mortality.
Leurs et al., 2010 [50] (Netherlands)	Data missing	No relationship between water hardness and ischemic heart disease mortality.

**Table 2:** Studies included in the meta-analysis.

Study	Years	Area	Water Hardness (CaCO_3_ mg/L)	Adjustments
Nagy et al. (2019) [38]	2000–2010	Hungary; 5 wine regions Szekszárd-Villány, Eger, Balaton, Tokaj, Hódmezővásárhely	Szekszárd-Villány: 294.2, Eger: 194.9, Balaton: 249.2, Tokaj: 138.6 CaO mg/L, Hódmezővásárhely: 81.90 CaO mg/L	Age-adjusted death rates, Socio-Economic Deprivation Index correlated with CVD
Nerbrandt et al. (2003) [39]	1989–1998	Sweden, Osthammar, Tocksfors	High: 167 CaCO_3_ mg/L Low: 45 CaCO_3_ mg/L (Calculated from medians of Mg^2+^ and Ca^2+^)	Asked for filtered water, mortality CVD/1000 inh
Rapant et al. (2020) [12]	1994–2008	Slovakia; Soft water region (34 municipalities) Hard water region (21 municipalities)	Hard water: 283.5 CaCO_3_ mg/L Soft water: 76.6 CaCO_3_ mg/L (Calculated from Mg^2+^ and Ca^2+^)	30 health indicators

## Data Availability

The data supporting the conclusions of this article are included within the article. The other datasets used and/or analyzed during the current study are available from the corresponding author upon reasonable request.
